# Extradural hematoma management: A case-control study with historical controls reassessing the 'zero mortality' goal

**DOI:** 10.1016/j.bas.2026.105963

**Published:** 2026-02-09

**Authors:** N. Marchesini, L. Rossi, E. Saiu, G. Pinna, F. Sala

**Affiliations:** a Leiden University Medical Center (LUMC), Leiden, the Netherlands; b Department of Neurosurgery, Azienda Ospedaliera Universitaria Integrata di Verona, Verona, Italy; c Department of Neurosciences, Biomedicine and Movement Sciences, University of Verona, Verona, Italy

**Keywords:** Traumatic brain injury, Extradural hematoma, Mortality, Outcomes

## Abstract

**Introduction:**

Extradural hematoma (EDH) remains a significant cause of mortality following traumatic brain injury (TBI), although fatality rates have declined. This study examines whether improvements in a regional trauma and neurosurgical care system over four decades have enabled the achievement of the long-standing goal of “zero mortality” in surgically treated EDH.

**Research question:**

Has the evolution of a regional trauma and neurosurgical care system over four decades reduced mortality and moved towards the ‘zero mortality’ goal in surgically treated extradural hematomas?

**Materials and methods:**

We retrospectively reviewed all patients who underwent surgery for EDH at our institution between 2011 and 2021. Demographic, clinical, and radiological data were collected and compared in a case-control design with a historical cohort treated at the same center in the early 1980s, when the “zero mortality” concept was first proposed.

**Results:**

Among 85 contemporary cases, most patients were aged 41–61 years (previously 11–20), and falls were the leading cause of injury (55% vs. 50% due to road traffic accidents; p = 0.007). Direct presentations increased (65% vs. 33%; p < 0.001), and 96% arrived within 6 h (vs. 65%; p < 0.001). Surgery within 6 h occurred in 68% of cases. Referred patients experienced significantly longer delays. Craniotomy was performed in 94% of cases. In-hospital mortality was 2.4% (vs. 4.7%).

**Discussion and conclusions:**

Although system-wide improvements have reduced mortality, the “zero mortality” target remains unmet. Delays in referral and triage continue to pose challenges, particularly in complex or rapidly evolving cases.

## Highlights

• Comprehensive retrospective study reassessing extradural hematoma mortality rates.
• Analysis reveals updated management strategies improving patient outcomes.
• Highlights importance of early diagnosis and timely treatment in reducing mortality.
• Uses contemporary data to challenge the ‘zero mortality’ treatment goal.

## List of abbreviations

TBI: Traumatic Brain Injury
EDH: Extradural Hematoma
ED: Emergency Department
GCS: Glasgow Coma Scale
GOS: Glasgow Outcome Scale
RTA: Road Traffic Accident
CT: Computed Tomography
ICU: Intensive Care Unit

## 1 Background

Extradural hematoma (EDH) of the head is a neurosurgical condition that may require urgent surgical intervention to treat or prevent neurological deterioration and reduce the risk of death following traumatic brain injury (TBI). Because the blood collection occurs outside the brain (extracerebral) and brain damage primarily results from increased intracranial pressure and herniation, timely clot removal and subsequent decompression of the brain parenchyma have been proposed as potentially life-saving measures for all patients. (Bricolo and Pasut, 1984), (Mendelow and Teasdale, 1983). Indeed, mortality rates have significantly decreased due to advancements in professional pre-hospital systems, triage, imaging techniques, and modern surgical and neurocritical management (Bir et al., 2015; Ruff et al., 2013; Cheung et al., 2007; Cordobes et al., 1981; Servadei et al., 1988; Baykaner et al., 1988). However, studies still report significant fatality rates even in well-established healthcare systems (Kulesza et al., 2021; Vilcinis et al., 2021; Gutowski et al., 2018; Clark et al., 2022).

An historic prospective data collection conducted in the early 1980s at our Institution documented an exceptionally low fatality rate during that period (approximately 5%), while deaths in contemporary series settled in the region of 20% (Bricolo and Pasut, 1984), (Cordobes et al., 1981), (Kvarnes and Trumpy, 1978; Heiskanen, 1975; Gennarelli et al., 1982; Reale et al., 1984). At that time, the authors hypothesized that achieving a “zero mortality” goal for EDH would be possible in the future, primarily through reduced intervention times.

While EDH has been extensively studied, few analyses have re-examined the feasibility of a “zero mortality” benchmark using modern surgical workflows and emergency system performance indicators. Specifically, to our knowledge, no recent studies have longitudinally compared outcomes and, more importantly, detailed care pathway metrics (e.g., door-to-surgery, referral patterns) against a historical baseline from the same institution over such an extended timeframe. This analysis provides a unique assessment of how trauma system maturation translates into measurable clinical process improvements and where persistent bottlenecks lie.

Four decades after the publication of that landmark paper, the objectives of the current study were: (i) to analyze and compare demographic and epidemiological changes in EDH within the same geographic area; (ii) to describe the evolution of the care system for this condition, focusing specifically on intervention times and their potential impact on clinical care and outcomes; (iii) to examine whether these contextual changes have modified the short term outcomes of patients undergoing surgery for an EDH, particularly in terms of in-hospital mortality.

## 2 Methods

### 2.1 Evolution of the neurotrauma system in the region

Our Hospital is located in a province in northeastern Italy, near Lake Garda, with approximately 775,000 inhabitants in 1982 (Popolazione residente ricostruita). At that time, the neurosurgical facility served not only this population but also additional patients from surrounding provinces that lacked neurosurgical care.

In the early 1980s, patients could reach our hospital directly, either via a non-centralized ambulance service or through referrals from peripheral hospitals. Upon arrival, all trauma patients, including those with polytrauma, were comprehensively managed by the neurosurgical team. After triage in the Neurosurgical Emergency Room, the on-call neurosurgeon determined the necessary examinations and decided on the appropriate course of action. If immediate surgery was required, a 24-h operating theater was available and promptly activated. Alternatively, patients were admitted to an intensive care unit managed by neurosurgeons.

In 1987, a centralized pre-hospital infrastructure was established, incorporating a widespread network for managing all medical emergencies in the province. Currently, the system includes approximately 30 emergency vehicles (including air transport), staffed by personnel with varying levels of expertise and equipped with all necessary resources for both routine and exceptional emergencies. In the following years, specific triage criteria—based on clinical, anatomical, and situational factors—were also introduced to determine the most appropriate hospital for patient transport and the optimal means of transportation. (Dettaglio Deliberazione della Giunta Regionale).

Nowadays, patients can access neurosurgical consultation either by directly presenting to our Hospital (a Level III facility) or through neurosurgical teleconsultation from one of five peripheral hospitals (Level II facilities) equipped with an Emergency Department (ED) and CT scan, with the farthest hospital located approximately 50 km away. In the first scenario, patients are triaged by a specialized ED nurse and evaluated by an ED physician, who determines the necessary diagnostic examinations. Based on clinical and CT findings, a specialist consultation, such as neurosurgical evaluation, may be requested. In cases of severe trauma, a dedicated trauma team is available, a whole-body CT scan is generally performed, and neurosurgeons are promptly notified. For referred patients, the on-call neurosurgeon remotely assesses the clinical scenario as described by the referring ED physician, reviews imaging findings, and determines whether the patient requires transfer based on specific criteria (Dettaglio Deliberazione della Giunta Regionale). If transfer is necessary, the patient may be transferred to our ED for further evaluation before hospitalization and, if needed, can access an operating room with on-call staff readily available. If intensive care is required, patients are admitted to a specialized neuro-ICU, where neurosurgeons provide consultations upon request. Currently, our neurosurgical service remains the only one in the province, offering 24-h urgent care to a population of nearly 930,000 inhabitants, in addition to approximately 5 million tourist arrivals annually, according to pre-pandemic data. (Regionea), (Regioneb).

### 2.2 Data collection and analysis

The current series includes 85 patients who underwent surgery for an EDH of the head at our tertiary-level institution between December 2011 and July 2021. Patients of all ages and with TBI of any severity were included. All patients had a CT-confirmed diagnosis of EDH, which was surgically verified and considered the primary intracranial lesion necessitating intervention and the only of clinical significance, following the approach used in the reference study.^1^

We conducted a retrospective analysis of medical records, radiological reports, and surgical reports. The collected data included demographics, clinical variables (pupillary status, Glasgow Coma Scale (GCS) on admission and before surgery, Glasgow Outcome Scale (GOS) at discharge, need for intensive care unit (ICU) admission, length of hospital stay, and discharge setting), as well as surgical details. The mechanism of injury was classified into falls (from standing height or greater), road traffic accidents (involving cars, pedestrians, motorcycles, bicycles, or other vehicles), and assaults. Patients with polytrauma or additional intracranial injuries were excluded if those lesions were deemed to significantly influence outcome or be the primary driver of neurological deterioration. This was done to isolate the effect of EDH and surgical timing. Quantitative hematoma volumetry was not routinely performed in clinical reports for all patients and was therefore not available for analysis. We calculated the following time intervals: (i) trauma-to-door time (from the accident to arrival at our hospital's emergency room); (ii) door-to-surgery time (from arrival at our hospital's emergency room to the start of the operation); (iii) trauma-to-surgery time (from the accident to the beginning of the operation). We further categorized the data based on the type of admission (direct admission, referring to patients who either directly presented or were directly transported to our emergency department; referred, referring to patients transferred to our hospital after evaluation at a peripheral hospital) and the degree of urgency (urgency, indicating that the operation was decided following the initial clinical and radiological evaluation; deferred urgency, indicating that the operation was decided after clinical and/or radiological deterioration).

When feasible, we compared the data in a case-control framework with those from a previous study that prospectively enrolled 107 patients who were admitted and operated on between 1980 and 1982 at the same institution.^1^ Given the sample size and data availability, formal matched-pair analysis was not performed. Instead, outcomes are presented for the overall cohorts and stratified by major prognostic factors (e.g., GCS, age) to facilitate a more nuanced comparison. Furthermore, patients in the contemporary cohort were classified as having ‘pure’ EDH (no associated cerebral lesion) or ‘mixed’ EDH (with associated lesions) based on admission CT imaging.

We assessed the equality of means for continuous variables using the *t*-test and employed Pearson's chi-square test to evaluate associations in frequency tables. Statistical analysis was performed using commercially available software (Microsoft Excel). A p-value of <0.05 was considered statistically significant.

## 3 Results

### 3.1 Demographics, clinical data and management

Table 1 summarizes the demographics and general characteristics of the 85 patients included in the current study, compared with the available data from the previous series.^1^ The majority of patients were male (67; 79%), with a mean age of 39.8 years (range 1-86), and the most represented age group was 41 to 60 years (28; 33%). The most common mechanism of injury was a fall (47; 55%), followed by road traffic accidents (RTAs) (24; 28%), with bicycle accidents being the most frequent (10; 13%). Most admissions were direct from the scene of trauma (55; 65%), while the remaining were referred cases. All referred cases already had a CT diagnosis of EDH. The majority of hematomas were predominantly parietal (30; 35%), and an associated skull fracture was found in 79 cases (93%).

**Table 1:** Demographics and trauma data of the current study and compared with those of the previous series of extradural hematomas of the head. n.a. = information not available.

Demographics and general characteristics			
	Current study, N (%)	Previous study, N (%)	P value
Number of patients	85 (100)	107 (100)	
Gender			
Males	67 (79)	89 (83)	>0.05
Females	18 (21)	18 (17)	
Age			
Mean (range)	39.8 years (1-86)	35 years (1-75)	
≤10	8 (9)	6 (6)	
11-20	11 (13)	39 (36)	
21-40	25 (29)	27 (25)	
41-60	28 (33)	25 (23)	
≥61	13 (15)	10 (9)	
0-40	44 (52)	72 (67)	**0.028**
>41	41 (48)	35 (33)	
Mechanism of injury			
Fall	47 (55)	45 (42)	**0.007**
Precipitation	23 (27)	n.a.	
From self-high	24 (28)	n.a.	
Road traffic accident (RTA)	24 (28)	53 (50)	
Bicycle	10 (13)	n.a.	
Car	6 (7)	n.a.	
Pedestrian	5 (7)	n.a.	
Motorcycle	2 (2)	n.a.	
Another vehicle	1 (1)	n.a.	
Assault	4 (5)	1 (1)	
Unknown	0 (0)	8 (7)	
Other	10 (12)	n.a.	
Admission			
Direct	55 (65)	35 (33)	**<0.001**
Referred from another hospital	30 (35)	72 (67)	
CT diagnosis available	30 (100)	22 (21)	
Site of hematoma			
Parietal	30 (35)	25 (23)	
Temporal	24 (28)	53 (50)	
Frontal	23 (27)	22 (21)	
Occipital	5 (6)	3 (3)	
Posterior cranial fossa	3 (4)	4 (4)	
Skull fracture	79 (93)	102 (95)	
Vault fracture	75 (88)	n.a.	
Base fracture	39 (46)	n.a.	
Associated cerebral lesion	41 (48)	26 (24)	
Degree of urgency			
Urgency	63 (74)	n.a.	
Deferred urgency	22 (26)	n.a.	

Neurological data are summarized in Table 2 and, when possible, compared to the available data from the previous series. At the scene of the accident and after resuscitation, most patients had a GCS of 13-15 (55; 65%) while 20 (24%) were comatose (GCS ≤8). Fifty-nine (69%) patients arrived at our hospital without sedation or intubation, and of these, 51 (86%) had a GCS score between 13 and 15. Nineteen cases (22%) experienced neurological deterioration after arriving at our hospital. The last GCS score before surgery was 13-15 for most cases (45; 53%) while in 24 cases (28%) it was ≤8 and 2 (2%) patients underwent surgery with dilated and not reactive pupils.

**Table 2:** Neurological data and management in the current study are compared with those from the previous series, when available. In the previous study, the initial neurological assessment was conducted upon admission to our institution, and the severity of the head injury was categorized based on GCS scores (3-4; 5-7; 8-15). The data from the current series are reported using both the previous and the current classification of head injury severity (3-8; 9-12; 13-15). ∗Percentages are based on patients who arrived without sedation or intubation; ∗∗Corresponds to the last available GCS. In the current study, the GOS was evaluated at discharge, while in the previous study, it was assessed at 6 months.

Neurological data and management			
	Current study, N (%)	Previous study, N (%)	P value
GCS on the scene after resuscitation			
3-8	20 (24)	n.a.	
9-12	10 (12)	n.a.	
13-15	55 (65)	n.a.	
Sedation and intubation status on admission			
Not sedated nor intubated	59 (69)	n.a.	
Sedated and intubated	26 (31)	n.a.	
Neurological worsening	19 (22)	12 (11)	**0.03**
GCS on admission (current classification)			
3-8	0 (0)∗	n.a.	
9-12	8 (14)∗	n.a.	
13-15	51 (86)∗	n.a.	
GCS on admission (previous classification)			
3-4	0	4 (4)	
5-7	0	32 (30)	
8-15	59 (100)∗	71 (66)	
GCS at operation (current classification)			
3-8	24 (28)∗∗		
9-12	16 (19)∗∗		
13-15	45 (53)∗∗		
GCS at operation (previous classification)			
3-4	11 (13)∗∗	10 (9)	
5-7	8 (9)∗∗	34 (32)	
8-15	66 (78)∗∗	64 (60)	
Pupillary status			
One dilated pupil	15 (18)	8 (8)	
Both pupils dilated and fixed	2 (2)	1 (1)	
Type of operation			
Craniotomy	80 (94)	70 (65)	**<0.001**
Craniectomy	5 (6)	37 (35)	**<0.001**
Intracranial pressure			
Not monitored	83 (98)	107 (100)	
Monitored	2 (2)	0 (0)	
Post-operative ICU	73 (86)	107 (100)	
GOS			
Good recovery/moderate disability	72 (85)	95 (89)	
Severe disability/vegetative state	11 (13)	7 (7)	
Dead	2 (2)	5 (5)	

Craniotomy with flap repositioning was the most common surgical procedure (80; 94%) and five patients (6%) underwent decompressive craniectomy. Intracranial pressure monitoring devices were placed in two patients (2%). Seventy-three patients (86%) received temporary treatment in the ICU postoperatively, with a median stay of 3 days (range 1-77). The overall length of hospital stay was 8 days (range 3-77) and it was significantly longer for patients with a last GCS score of 3-8 compared to those with a GCS score of 9-15 (20 days vs 7 days; p = 0.0002).

### 3.2 Time intervals

Table 3 compares timing intervals to those of the previous series and Supplement 1 summarizes them in more detail.

**Table 3:** Time intervals from trauma to door and from trauma to surgery in the current series as compared with the historical cohort.

	Current study	Previous study	P value
Trauma-to-door			
**<6 h**	65 (96)	70 (65)	**<0.001**
**<24 h**	67 (99)	86 (80)	**<0.001**
**>24 h**	1 (1)	21 (20)	**<0.001**
**Trauma-to-surgery**			
**<6 h**	48 (68)	61 (57)	>0.05
**7**–**24 h**	19 (27)	30 (28)	>0.05
**>24 h**	4 (6)	16 (15)	0.053

The median trauma-to-door timing was 1.4 h (0.2-26.7), door-to-surgery was 2.3 h (0.5-132.3), and trauma-to-surgery 4.9 h (1.4-119.4). Overall, the majority of cases (96%) arrived at our facility within 6 h from the accident, and 68% underwent surgery within 6 h of injury.

There was no significant difference in the median trauma-to-door time between urgent and deferred cases (1.4 h vs 1.8 h). However, urgent cases had shorter door-to-surgery (1.8 h vs 9.2 h) and trauma-to-surgery (3.8 vs 9.8 h) intervals (p < 0.00001).

Differences were observed between direct admissions and referred cases. In urgent cases, the median trauma-to-door time was significantly longer for referred patients compared to direct admissions (3.7 h vs 1.2 h; p = 0.00079). Consequently, referred cases had longer trauma-to-surgery intervals (5.2 h vs 3.4 h; p = 0.028), although they had shorter door-to-surgery timing (1.2 h vs 2.1 h; p = 0.014). In deferred urgencies, the median trauma-to-door time was significantly longer for referred cases compared to direct admissions (4.6 h vs 1.1 h; p < 0.001). However, there were no differences between direct and referred cases in the door-to-surgery (9.7 h vs 8.8 h; p > 0.05) and trauma-to-surgery (12.1 h vs 8.5 h; p > 0.05) intervals.

Overall, cases arriving at our hospital during the day shift (8 a.m. to 8 p.m.) had shorter trauma-to-door intervals than those arriving during the night shift (8 p.m. to 8 a.m.) (1.2 h vs 2.9 h; p = 0.015), and this difference was close to the significance threshold even for urgent cases (1.2 h vs 2.8 h; p = 0.052). The median door-to-surgery timing was not different between day and night arrivals, even for urgent cases (1.6 h vs 1.9 h; p > 0.05).

There were no significant differences in timing intervals between cases with a last GCS score of 3-8 and those with a GCS score of 9-15.

### 3.3 Outcomes

Table 4 summarizes the neurological outcomes at discharge. Overall, 83 patients (98%) were alive and 72 (85%) showed a favorable outcome (good recovery or moderate disability). The rate of favorable outcomes was higher in the groups with a last GCS score of 13-15 or 9-12 compared to 3-8 (98% *and* 94% *vs* 54%; p = 0.007 and p < 0.00001). On the other hand, 38% of patients with a GCS score of 3-8 had unfavorable outcomes (severe disability or vegetative state), and 82% (n = 9) of patients with poor outcomes had a GCS score of 3-8.

**Table 4:** Outcome at discharge in relation to last GCS score, timing, urgency, admission. GR = good recovery; MD = moderate disability; SD = severe disability; VS = vegetative state.

Overall data	N (%)	GR/MD (%)	SD/VS (%)	Dead (%)
	85 (100)	72 (85)	11 (13)	2 (2)
**Last GCS**				
**3**–**8**	24 (28)	13 (54)	9 (38)	2 (8)
**9**–**12**	16 (19)	15 (94)	1 (6)	0 (0)
**13**–**15**	45 (53)	44 (98)	1 (2)	0 (0)
**Neurological worsening**				
**Yes**	19 (22)	15 (79)	3 (16)	1 (5)
**No**	66 (88)	57 (86)	8 (12)	1 (2)
**Pupillary status**				
**Normal**	68 (80)	62 (91)	6 (9)	0 (0)
**One dilated**	15 (18)	10 (67)	4 (27)	1 (7)
**Both dilated**	2 (2)	0 (0)	1 (50)	1 (50)
**Age**				
**<50**	58 (68)	53 (91)	4 (7)	1 (2)
**≥50**	27 (32)	19 (70)	7 (26)	1 (4)
**Associated cerebral lesion**				
**Yes**	41 (48)	32 (78)	8 (20)	1 (2)
**No**	44 (52)	40 (91)	3 (7)	1 (2)
**Trauma-to-surgery (71)**				
**<4 h**	29 (41)	22 (76)	7 (24)	0 (0)
**>=4 h**	42 (59)	37 (88)	3 (7)	2 (5)
**<5 h**	36 (51)	29 (81)	7 (19)	0 (0)
**>=5 h**	35 (49)	30 (86)	3 (9)	2 (6)
**<6 h**	45 (63)	36 (80)	8 (18)	1 (2)
**>=6 h**	26 (37)	23 (88)	2 (8)	1 (4)
**Urgency**				
**Urgency**	63 (74)	54 (86)	7 (11)	2 (3)
*Direct adm*	42 (67)	34 (81)	7 (17)	1 (2)
*Referred*	21 (33)	20 (95)	0	1 (5)
**Deferred urgency**	22 (26)	18 (82)	4 (18)	0 (0)
*Direct adm*	13 (59)	10 (77)	3 (23)	0 (0)
*Referred*	9 (41)	8 (89)	1 (11)	0 (0)
**Admission**				
**Direct**	55 (65)	44 (80)	10 (18)	1 (2)
**Referred**	30 (35)	28 (93)	1 (3)	1 (3)
**Arrival**				
**8 a.m. to 8 p.m.**	46 (54)	38 (83)	8 (17)	0 (0)
**8 p.m. to 8 a.m.**	39 (46)	34 (87)	3 (8)	2 (5)

Based on imaging findings, 44 patients (52%) were classified as having ‘pure’ EDH and 41 (48%) as ‘mixed’ EDH with associated cerebral lesions. The mortality rate was similar between the two groups (1/44, 2.3% in ‘pure’ EDH vs. 1/41, 2.4% in ‘mixed’ EDH). However, the rate of favorable outcome (GOS GR/MD) was higher in the ‘pure’ EDH group (40/44, 90.9% vs. 32/41, 78.0%; p = 0.11), while unfavorable outcomes (SD/VS) were more frequent in the ‘mixed’ EDH group (8/41, 19.5% vs. 3/44, 6.8%).

Nineteen patients (22%) experienced neurological deterioration after reaching our hospital, but the rate of favorable or unfavorable outcomes did not differ from those who did not deteriorate. Patients who underwent surgery with one dilated and not reactive pupil had higher rates of unfavorable outcomes (27% *vs* 9%; p = 0.011). Two patients had both dilated and not reacting pupils: one died, and one was discharged with severe residual disability. Patients younger than 50 years had significantly higher rates of favorable outcomes compared to older patients (91% *vs* 70%; p = 0.012), while the rate of unfavorable outcomes was higher in patients older than 50 years (26% *vs* 7%).

No significant differences in the rate of favorable or unfavorable outcomes were found in patients operated on during the examined time intervals (<4 h *vs* ≥ 4 h; <5 h *vs* ≥ 5 h; <6 h *vs* ≥ 6 h), but both deaths occurred in patients operated on more than 4 h after trauma. No differences were found between direct and referred admissions, urgent and deferred cases, and day and night arrivals.

Two fatalities occurred, resulting in a mortality rate of 2.4%. In one case, an 85-year-old man with several comorbidities presented to our hospital 4 h after trauma with a GCS score of 11 and deteriorated to 8 before intubation and surgery, which started approximately 6 h after the trauma. The patient died on the 25th postoperative day in the ICU due to pulmonary complications. The other fatality involved a young man who presented to a peripheral hospital about 1 h after TBI. He rapidly deteriorated to a GCS score of 3 and reached our facility approximately 3 h after the trauma, intubated, but with both pupils dilated and non-reactive. Surgery started promptly after arrival, including a craniotomy and placement of an ICP probe, but the patient died on postoperative day 7.

## 4 Discussion

The current study provides an overview of the changes observed in the demographics of patients operated on for an EDH in the same geographic area over four decades. A trend toward greater involvement of females and older patients was noted, along with an increase in falls as the primary mechanism of injury, while RTAs decreased. Additionally, insights were gained regarding the impact of advancements in the trauma system on short-term outcomes: more patients were directly transferred to the definitive care center, pre-hospital airway protection of comatose patients became routine, and surgeries were performed more promptly with improved preoperative neurological status. In-hospital fatalities were further reduced, yet achieving zero mortality remains an unrealized goal. Although EDH is a well-studied surgical emergency and surgical technique has substantially remained the same, the context surrounding surgery has transformed dramatically. This study focused on evaluating the impact of these systemic changes—including pre-hospital triage, imaging availability, referral coordination, and operative logistics—on the pathway to surgery and subsequent outcomes. In other words, this study contributes a rare longitudinal perspective, uniquely positioned to assess how changes in trauma systems, prehospital care, and operative logistics influence outcomes. By directly comparing two cohorts treated 40 years apart at the same center, we identify persistent challenges and measurable progress in pursuit of the long-held goal of zero mortality.

EDH of the head remains the most common surgical indication for patients with TBI in various regions of the world. As observed in other well-established systems, our study confirmed a significant decline in surgically treated EDHs over the years. Demographic changes and safety policies have contributed to a shift towards other types of intracranial post-traumatic hemorrhages, while a decrease in the overall incidence of TBI in Europe has not yet been demonstrated (Aromatario et al., 2021; Capizzi et al., 2020; Peeters et al., 2015).

In our context, three policies introduced after the publication of the first series could have played a pivotal role. Firstly, in 1986, a national law mandated helmet use for motorcyclists of all ages and for moped riders up to 18 years old. After the law went into effect, helmet use increased from 15% to 97% and has remained stable since then. In 2000, a new helmet law came into force, extending the obligation to adults riding mopeds. A study comparing emergency room visits before and after the law showed a 40% decrease in emergency room arrivals. Specifically for TBI, arrivals decreased by 75%, and hospitalizations in neurosurgery departments decreased by 79% (Sorveglianze nazionali Impatto di una, 2000). The second safety policy was introduced in 1988, making seat belt use mandatory for front-seat passengers in vehicles. In 2003, this law was extended to include rear-seat passengers as well (La normativa, 2022). Although there were regional differences, after the introduction of the law, the rate of regular seat belt use increased from 11% in the 1980s to approximately 85% in the front seats and 30% in the rear seats (95% and 43% in our region, respectively) (EpiCentroa). Thirdly, after 1992, various traffic laws were introduced to limit drunk driving. Nowadays, blood alcohol levels in drivers older than 21 years can't exceed 0,5 g/l, while in younger individuals, any levels above 0,0 g/l are not allowed, and sanctions are progressively more severe with higher blood levels (Salute). Between 2000 and 2021, such laws have contributed to an overall 42% reduction in RTAs with injuries to individuals, along with a parallel reduction in deaths from these accidents by over 60% (Statistiche Istat), (EpiCentrob). In our series, the significant reduction in the number of injuries due to RTAs may be correlated with an overall decrease in head injuries and EDHs due to this mechanism, with a consequent increase in the ratio of injuries due to other causes, especially falls in elderly patients.

The development of pre-hospital systems has been associated with more favorable outcomes for patients with TBI (Nielsen et al., 2012; Meena et al., 2018; Denninghoff et al., 2017). However, the presence of trained personnel and dedicated infrastructures is relatively recent and remains absent or inadequate in many regions worldwide, with significant effects on the efficiency and effectiveness of care (Clark et al., 2022), (Nielsen et al., 2012), (Meena et al., 2018). In the context of the first half of the 1980s when the reference study was conducted, a single telephone number for health emergencies did not exist, and every city had a telephone number for their ambulance service. There was therefore no coordination and no territorial operations center. The main goal was the rapid transport of the patient to the nearest hospital (“scoop and run”) with minimal action on the scene, diagnostic work-up, stabilization, and treatment. It was not rare for those with access to a car to take the patient directly to the hospital. This was chosen based on proximity criteria, and patients often had to be transferred to the appropriate facility, resulting in a considerable increase in management timing (La storia del 118). The current evidence suggests that the direct transport of TBI patients to the definitive center of care may improve outcomes, and the pre-hospital systems have evolved accordingly (Harrington et al., 2005). Comparing the results of this new series with those of the reference study, the number of cases directly transferred to the definitive center of care doubled, and a remarkable reduction in trauma-to-door timing was observed. This, in turn, reduced trauma-to-surgery intervals and allowed a higher rate of cases to reach surgery with a better neurologic status. The longer pre-hospital intervals observed during the night shift could be partially explained by the unavailability of air transportation for night arrivals. Pre-hospital clinical practice has significantly evolved, and nowadays the concept that aggressive airway management improves outcomes of patients with severe TBI is generally accepted (Gamberini et al., 2019). Accordingly, none of the patients of the current series found comatose at the scene of the accident reached our hospital without airway protection.

Timeliness of treatment is considered a quality indicator for trauma care, and according to the American College of Surgeons Committee on Trauma, institutions performing craniotomies for extradural or subdural hematomas later than 4 h after ED admissions should be audited for performance improvement of care. Indeed, it has been shown that early clinical assessment and quick access to neurosurgical consultation and care reduce mortality and length of stay of these patients (Clark et al., 2022), (Kim, 2011; Haselsberger et al., 1988; Mendelow and Gillingham, 1979)(Kim, 2011; Haselsberger et al., 1988; Mendelow and Gillingham, 1979). Although pre-hospital intervals were significantly reduced, and our median door-to-surgery timing falls below this threshold of acceptance, we observed only a trend towards a shorter overall trauma-to-surgery interval compared to the reference study. We speculate that this could be at least partially due to the current model of early in-hospital management, a system that involves different intermediate stakeholders in the EDs, from nursing triage to medical consultation to imaging. This hypothesis could be supported by the shorter door-to-surgery intervals of referred patients, as such cases typically complete the diagnostic workup in the referring center. In other words, while the current model has improved the safety and accuracy of diagnosis on one side, its positive influence on overall efficiency seems to have been less impactful. Future studies should focus on identifying barriers that hinder timely triage and diagnosis in EDs.

Compared with the previous series, a higher number of cases deteriorated after arrival at our institution, and the reason could be twofold. Firstly, patients came to our attention earlier, and it's plausible that neurological worsening was intercepted in the hospital rather than the pre-hospital setting. Secondly, a cautious “wait and see” approach could have been chosen in some cases, involving strict clinical and radiological observation.

Craniotomy with flap repositioning represents the gold standard procedure for operative management of EDH nowadays, as the usefulness of routine decompressive craniectomy become questionable, even for comatose patients (Vilcinis et al., 2021), (Brain Trauma Foundation), (Bisen et al.). Accordingly, the rate of craniectomies significantly reduced compared to the reference study, in which one-third of cases underwent decompression according to institutional policy. Out of the five decompressive craniectomies performed, three were in the posterior fossa, and the technique was chosen based on the surgeon's preference. As part of our department's routine practice, a small exploratory dural incision is made to rule out subdural bleeding after removing the extradural clot. In two comatose patients, unexpected brain bulging was observed upon dural opening, and the surgeon opted not to reposition the bone flap for safety reasons.

Perioperative care has also evolved in parallel with new evidence. For example, while in the previous series all patients received steroids for 3-4 days after surgery, current guidelines recommend against administering steroids in TBI cases. Similarly, the use of hypertonic therapies is now restricted to selected comatose cases (Carney et al., 2017).

The comparison of neurological outcomes between the two series is somewhat biased due to methodological issues. While the previous study evaluated outcomes up to 6 months after surgery, our data only provide information up to discharge. However, our results overall confirm what has been previously demonstrated: younger patients and those operated on with a better neurological status show better neurological outcomes. Moreover, patients with associated cerebral lesions had a notably, though not statistically significant, lower rate of favorable outcomes and a higher rate of severe disability compared to those with ‘pure’ EDH. This underscores that associated intracranial injuries signify a more severe brain insult, where the prognosis is influenced by the combined burden of the epidural collection and the underlying parenchymal injury. It should also be noted that the neurological status of patients in the previous study was generally better at follow-up. However, this difference can be easily explained by the longer time those patients had for recovery before assessment.

On the other hand, data regarding our primary outcome of interest, which is in-hospital mortality, can be appropriately compared. The previous series reports a total of five deaths (4.7%), with two of these due to pulmonary postoperative complications after emerging from the coma. Of the remaining three cases, two deaths were attributed to delays in referral, and one was attributed to delays in the diagnostic process at our facility.

In the current series, two patients died (2.4%). For the older patient, the main cause could be attributed to late presentation to the hospital with an already compromised neurological status. It is well known that the mortality rate due to TBI is significantly higher in the elderly and in those with a preoperative GCS lower than 9 (Réa-Neto et al., 2023), (Launey et al., 2022). Indeed, in the contemporary cohort, mortality was 8% (2/24) in patients with a last GCS of 3-8, and 0% (0/61) in those with GCS 9-15. Similarly, in the historical cohort, based on admission GCS, no deaths occurred among patients reaching surgery with a GCS of 8 or better. Population-based educational policies could be helpful in raising awareness about TBIs in the elderly and prompt medical consultation in specific situations. In the second fatal case, the patient was correctly triaged at the level II facility according to the criteria mentioned above (Dettaglio Deliberazione della Giunta Regionale). However, the rapid neurological deterioration was unpredictable, and unavoidable logistical delays in referrals only allowed surgery to be attempted as a desperate but futile measure. Current triage tools may fail to timely detect potentially evolving intracranial bleedings, which can affect patient referrals (Alqurashi et al., 2022). For this reason, several ongoing efforts are addressing the need for prompt diagnosis of cerebral hematomas after TBI (El et al., 2021; Kontojannis et al., 2019; Anderson et al., 2020).

## 5 Conclusions

Over the span of four decades, the evolution of the care system for patients undergoing surgery for EDH has led to a further reduction in the rate of in-hospital fatalities. However, the targeted “zero-mortality” goal has not been attained. While an adjusted comparison is not possible, the observed reduction in mortality, particularly in the most severe cases, alongside significantly improved process times, suggests a meaningful positive evolution of the care system. However, with anticipated demographic shifts and the existing referral and triage tools, there remains a tangible possibility that a minority of cases may not receive timely surgical intervention. This study underscores the value of revisiting historical benchmarks using current real-world data to identify system-level gaps in emergency neurosurgical care.

## 6 Strengths and limitations

This study provides a rare longitudinal perspective on the evolution of EDH care over four decades within a single institution. A major strength lies in the ability to directly compare contemporary data with a historical cohort treated at the same center, allowing for a meaningful assessment of changes in epidemiology, pre-hospital triage, and surgical management. The focus on specific time intervals along the care pathway offers practical insights into system performance and areas for improvement.

However, several limitations must be acknowledged. As a single-center analysis from a specific region in northeastern Italy, our findings are inherently influenced by local demographics, pre-hospital logistics, and referral patterns. The aging population in this region, as noted, impacts injury mechanisms and outcomes. Therefore, the absolute mortality rates and time intervals may not be directly generalizable to other geographic or healthcare contexts. However, the longitudinal methodology and the focus on system-level changes offer a template for internal audit and highlight common challenges in achieving timely care, such as referral delays and triage efficiency, which are relevant to trauma systems globally.

The retrospective design is subject to the inherent risks of missing data, documentation variability, and selection bias. Specifically, a direct comparison of outcomes between cohorts is constrained by differences in data granularity and the lack of key variables (e.g., precise hematoma volume, detailed comorbidities) in the historical cohort, precluding advanced statistical adjustment such as propensity score matching. This limits causal inference regarding the impact of system changes on mortality reduction.

Time intervals were derived from clinical documentation and may be affected by inaccuracies or inconsistencies in record-keeping. Long-term follow-up data were not systematically collected across the study period due to lack of centralized outpatient tracking. The sample size, although covering a decade of surgical EDH cases, remains relatively small, which may reduce statistical power. The study focuses on in-hospital outcomes; long-term functional or neurological recovery was not assessed. Additionally, potential confounding factors—such as associated injuries, comorbidities, or variability in perioperative care—were not systematically controlled.

Importantly, patients treated after July 2021 could not be included due to a transition in the hospital's electronic medical record system. This change resulted in a loss of data granularity, particularly regarding time-sensitive variables essential for this analysis, and limited our ability to extend the study to more recent cases.

Despite these limitations, this study highlights significant trends in EDH management and underscores ongoing challenges in achieving optimal outcomes, especially in relation to timely triage and referral.

## Ethics statement

The study was conducted in accordance with the ethical principles of the Declaration of Helsinki. All data used in this study were de-identified to ensure confidentiality and to comply with data protection regulations.

## Consent for publication

Not applicable.

## Availability of data and materials

Anonymized data are available upon reasonable request to the corresponding author.

## Competing interests

The corresponding author (NM) and the senior author (FS) are serving, at the time of submission, in the Editorial Board of Brain and Spine.

## Authors' contributions

All authors have given substantial contributions to the conception and the design of the study, Nicolò Marchesini, Lorenzo Rossi and Edvige Saiu to acquisition, analysis and interpretation of the data. All authors have participated in drafting the manuscript. All authors read and approved the final version of the manuscript.

## Declaration of generative AI and AI-assisted technologies in the writing process

During the preparation of this work, the author(s) utilized ChatGPT to identify and correct English language errors. After using this tool/service, the author(s) reviewed and edited the content as needed and take(s) full responsibility for the content of the publication.

## Funding

This research received no specific grant from any funding agency in the public, commercial, or not-for-profit sectors.

## Declaration of competing interest

The authors declare the following financial interests/personal relationships which may be considered as potential competing interests:The corresponding author is serving, at the time of submission, in the Editorial Board of Brain and Spine as a Reviewer Board member If there are other authors, they declare that they have no known competing financial interests or personal relationships that could have appeared to influence the work reported in this paper.
