# Supplementary material for: Extradural hematoma management: A case-control study with historical controls reassessing the 'zero mortality' goal
Source: Brain Spine. 2026 Feb 9;6:105963. doi: 10.1016/j.bas.2026.105963 (PMC12917510; doi:10.1016/j.bas.2026.105963)
Supplement: Multimedia component 1 [file mmc1.docx]

**Detailed timing intervals expressed in hours**

|  | **Trauma-to-door** | **Door-to-surgery** | **Trauma-to-surgery** |
| --- | --- | --- | --- |
| **Total** (n=85) | Median 1.4  Mean: 2.5  Range: 0.2-26.7 | Median: 2.3  Mean: 8.6  Range: 0.5-132.3 | Median: 4.9  Mean: 9.1  Range: 1.4-119.4 |
| **Urgency** (n=63) | Median: 1.4  Mean: 2.5  Range: 0.2-26.7 | Median: 1.8  Mean: 2.3  Range: 0.5-13.2 | Median: 3.8  Mean: 5.0  Range: 1.4-28.3 |
| **Deferred urgency** (n=22) | Median: 1.8  Mean: 2.7  Range: 0.5-7.1 | Median: 9.2  Mean: 27.2  Range: 2.1-132.3 | Median: 9.8  Mean: 22.4  Range: 3.3-119.4 |
| **Direct admission** (n=55) | Median: 1.1  Mean: 1.4  Range: 0.2-5 | Median: 2.5  Mean: 9.6  Range: 0.8-132.3 | Median: 3.7  Mean: 7.8  Range: 1.4-119.4 |
| *Urgency* (n=42) | Median: 1.2  Mean: 1.4  Range: 0.2-5 | Median: 2.1  Mean: 2.7  Range: 0.8-13.2 | Median: 3.4  Mean: 4.1  Range: 1.4-18.3 |
| *Deferred urgency* (n=13) | Median: 1.1  Mean: 1.1  Range: 0.5-2.3 | Median: 9.7  Mean: 31.2  Range: 3.5-132.3 | Median: 8.5  Mean: 22.8  Range: 4.7-119.4 |
| **Referred** (n=30) | Median: 3.9  Mean: 4.9  Range: 0.8-26.7 | Median: 1.6  Mean: 6.6  Range: 0.5-67.4 | Median: 5.5  Mean: 11.6  Range: 2.8-71 |
| *Urgency* (n=21) | Median: 3.7  Mean: 5.1  Range: 0.8-26.7 | Median: 1.3  Mean: 1.4  Range: 0.5-4.1 | Median: 5.2  Mean: 6.8  Range: 2.8-28.3 |
| *Deferred urgency* (n=9) | Median: 4.6  Mean: 4.7  Range: 2.5-7.1 | Median: 8.8  Mean: 19.9  Range: 2.1-67.4 | Median: 12.1  Mean: 21.9  Range: 3.3-71 |
| **Arrival 8 AM to 8 PM** (n=46) | Median: 1.2  Mean: 1.6  Range: 0.2-5.0 | Median: 2.5  Mean: 10.4  Range:0.5-118.3 | Median: 4.5  Mean: 11.4  Range:1.4-119.4 |
| *Urgency* (n=32) | Median: 1.2  Mean: 1.5  Range: 0.2-5.0 | Median: 1.6  Mean: 2.4  Range:0.5-13.2 | Median: 3.3  Mean: 4.1  Range: 1.4-18.3 |
| *Deferred urgency* (n=14) | Median: 1.3  Mean: 2.0  Range: 0.5-4.6 | Median: 14.5  Mean: 27.4  Range: 3.5-118.3 | Median: 20.2  Mean: 29.8  Range: 4.7-119.4 |
| **Arrival 8 PM to 8 AM** (n=39) | Median: 2.9  Mean: 3.6  Range: 0.5-26.7 | Median: 2.0  Mean: 6.4  Range: 0.7-132.3 | Median: 5.1  Mean: 6.4  Range: 2.4-28.3 |
| *Urgency* (n=31) | Median: 2.8  Mean: 3.5  Range: 0.5-26.7 | Median: 1.9  Mean: 2.2  Range: 0.7-6.3 | Median: 4.9  Mean: 5.9  Range: 2.4-28.3 |
| *Deferred urgency* (n=8) | Median: 5.4  Mean: 4.3  Range: 1.0-7.1 | Median: 7.0  Mean: 26.8  Range: 2.1-132.3 | Median: 8.8  Mean: 8.9  Range: 7.5-14.3 |
| **Last GCS 3-8** (n=24) | Median: 1.3  Mean: 1.9  Range: 0.5-5.4 | Median: 2.1  Mean: 8.9  Range: 0.5-132.3 | Median: 3.7  Mean: 5.2  Range: 2.0-20.2 |
| **Last GCS 9-15** (n=61) | Median: 1.7  Mean: 2.8  Range: 0.2-26.7 | Median: 2.4  Mean: 8.5  Range: 0.5-118.3 | Median: 5.3  Mean: 10.8  Range: 1.4-119.4 |
